# Circulating long non-coding RNA TTTY15 and HULC serve as potential novel biomarkers for predicting acute myocardial infarction

**DOI:** 10.1186/s12872-022-02529-5

**Published:** 2022-03-04

**Authors:** Jiajia Xie, Wenjun Liao, Wuqi Chen, Disheng Lai, Qidong Tang, Yuhui Li

**Affiliations:** 1 Department of Cardiology, Guangdong Second Provincial General Hospital, Guangzhou, 510317 China; 2 Department of Cardiology, Guangdong Provincial People’s Hospital, Guangzhou, China

**Keywords:** Acute myocardial infarction, Diagnosis, lncRNA, TTTY15, HULC

## Abstract

**Introduction:**

Acute myocardial infarction (AMI) is a ubiquitous cardiovascular disease ensuing adverse prognosis caused by myocardial necrosis. Effective and rapid diagnosis of AMI is essential to following treatment in clinical practice while the existed biomarkers have inherent limitations. Consequently, exploration of novel biomarkers is needed. Long noncoding RNA (lncRNA) emerges as the upcoming biomarkers adopted in clinical use, and we aim at investigating the diagnostic power of lncRNA TTTY15 and HULC in AMI patients.

**Method:**

We measured lncRNA level in 80 AMI patients and 36 healthy volunteers in discovering cohort and 50 AMI patients and 20 healthy volunteers in verification cohort with quantitative RT-PCR method. Receiver operating characteristic (ROC) analysis was administered to detect the diagnostic power of selected lncRNAs. Regression and correlation analyses were performed to explore the related factors.

**Results:**

ROC analysis reveals the superiority of TTTY15 and HULC as biomarkers against conventional AMI biomarkers CKMB (AUC of TTTY15: 0.915 versus CKMB: 0.768 versus TnT: 0.869); AUC of HULC: 0.905 versus CKMB: 0.768 versus TnT: 0.869). Regression and correlation analysis indicates that TTTY15 and HULC may be one of the contributing factors to AMI and related to accepted risk factors.

**Conclusion:**

Our results revealed the diagnostic potency of lncRNA TTTY15 and HULC, and they could also be treated as novel therapeutic targets in AMI therapy, hinting inspiration to the cardiologist in clinical practice.

**Supplementary Information:**

The online version contains supplementary material available at 10.1186/s12872-022-02529-5.

## Introduction

Acute myocardial infarction (AMI), as a ubiquitous cardiovascular disease ensuing adverse prognosis, is caused by myocardial necrosis induced by unstable ischemic syndrome [1]. Contemporary treatment for AMI is percutaneous coronary intervention (PCI) or coronary artery bypass graft (CABG), with attendant thrombolytic therapy, respectively [2, 3]. Effective and rapid diagnosis of AMI is essential for the selection of a corresponding treatment strategy as it could prevent the progressively deleterious ravage to the myocardium and accrue considerable prognosis [4, 5]. Conventional biomarkers like cardiac troponin (cTns) and creatinine kinase MB (CKMB) are adopted as the golden standard in AMI diagnosis, yet the inherent limitations that other cardiovascular diseases may present exaggeration of cTns and CKMB level still exist [5, 6]. Consequently, exploration of novel biomarkers with adequate accuracy is imminent.

With the advanced development of genomic techniques, enormous biomarkers are prone to be applicable in the clinical diagnosis of AMI [7]. Specifically, with plausible RNA-sequencing techniques, long non-coding RNAs (lncRNA) of more than 200 nucleotides are proved to play a vital role in regulating gene expression by various mechanisms including gene transcription, translation, epigenetic inheritance, etc. [8, 9]. Moreover, plasma lncRNA level has been reported to be altered in diseases including the cardiovascular system, so leveraging lncRNA as diagnostic markers is feasible [10–12].

lncRNA highly up-regulated in liver cancer (HULC) is known as abnormal secretion in cancer cells and is reported to be protective against myocardial I/R injury and H/R cardiomyocyte apoptosis by inhibiting NLRP3/Caspase-1/1L-1β signaling pathway [13, 14]. lncRNA testis-specific transcript Y-linked 15 (TTTY15) is found to be upregulated in AMI patients and H2O2-stimulated myocardial cells, and the theory has been verified utilizing knockdown experiment [15]. Thus, HULC and TTTY15 are conceivable to be potential diagnostic biomarkers in AMI patients.

In this study, we aim at exploring the feasibility of adopting HULC and TTTY15 as novel biomarkers compared with CKMB in AMI diagnosis.

## Method

### Participants

We enrolled all AMI patients presented to Guangdong Second Provincial General Hospital (Guangzhou, China) from September 2020 to November 2021 and a total of 80 AMI patients were enrolled with 36 recruited healthy controls. A verification cohort with a total of 50 AMI patients and 20 controls were recruited from Guangdong Provincial People’s Hospital to verify the results. All enrolled patients were negative for COVID-19. Inclusion criteria were AMI diagnosis conforming to 2017 ESC guideline [16], elevated conventional cardiac biomarkers above the upper limit, and abnormal echocardiogram (ECG) findings. Exclusion criteria were patients complicated with other advanced or serious diseases such as malignancy and organ failure. Healthy controls were volunteers without a history of cardiovascular diseases or other organ issues. Conventional biomarkers, CKMB and troponin-T (TnT), were measured routinely in the first-meet. For the hypothetic biomarkers, the lncRNAs were measured just after admission (pre-PCI) and after the PCI procedure in the AMI group, while they were measured just after recruiting in healthy controls.

To verify the diagnostic value of selected lncRNA, another cohort with a total of 50 AMI patients receiving PCI procedure and 20 controls were recruited in another center, Guangdong Provincial People’s Hospital. Conventional biomarkers and the selected lncRNA were measured as above.

### Blood sample collection

Regarding the AMI group, peripheral blood samples were obtained just after admission (pre-PCI) and after the PCI procedure, while for healthy controls, blood samples were obtained after recruiting. All blood samples were collected in the plain tube (BD Vacutainer®, 369714) containing EDTA anticoagulant without coagulation and hemolysis. The collected blood samples were centrifuged at 3500 rpm for 10 min, the supernatant was carefully transferred into an RNase-free tube and then immediately frozen at − 80 °C. Since the two IncRNAs are also expressed in blood cells, sample processing is carried out immediately after qualified samples are collected to minimize the contamination caused by the death and destruction of blood cells.

### RNA isolation and Quantitative RT-PCR

Total RNA was extracted from plasma samples using Plasma/Serum RNA Purification Maxi Kit (Norgen, Product #56200) as described by the manufacturer. iScript® cDNA Synthesis Kit (Bio-Rad) was adopted to perform reverse-transcription of cDNA (component: total 20 μl reaction system containing 200 ng RNA template, 4 μl 5 × iScript Reaction mix, 1 μl iScript Reverse Transcriptase, and Nuclease-free water; reaction protocol: 5 min at 25 °C, 30 min at 42 °C, 5 min at 85 °C, and then hold at 4 °C). RNase-Free DNase I Kit (Norgen, Product #25710) was adopted to on-colomn DNA removal process to avoid genomic DNA contamination as described by the manufacturer. Green PCR Kit (Takara, Dalian, China) was administered in qRT-PCR procedure with specifically designed primers for lncRNA HULC and TTTY15. When performing qRT-PCR of HULC and TTTY15, GAPDH was treated as the internal control. Specific primers used in this study are as follows: HULC: forward 5ʹ-ATGGGGGTGGAACTCATGATGG-3ʹ, reverse 5ʹ-AAGAATGGACATCATTT ATTTCA-3ʹ; TTTY15: TTTY15, forward 5′-TGAGGGAGGGATGTAGCTTT-3′, reverse 5′-GAAGTCAAGCAGGCAACTGA-3′; GAPDH: forward 5ʹ-TGCACCACCAACTGCTTAGC-3ʹ, reverse 5ʹ-GGCAT GGACTGTGGTCATGAG-3ʹ. The relative expression level of detected lncRNA was measured following 2^−ΔΔcq^ methods. Boxplot showing the cq value of the selected lncRNA was displayed in Additional file 1: Figure S1.

### Statistical analysis

All data were presented with mean ± SD or number (percentage), and involved data analyses were performed in SPSS 23.0 and R (Version 3.6.2) software. Independent sample t-test, spearman correlation test, and chi-square test were performed in SPSS. The receiver operating characteristic (ROC) curve was employed to evaluate the specificity and sensitivity of selected lncRNA and the area under the ROC curve (AUC) was used to evaluate the predictive power. Both ROC and AUC were obtained via R with the pROC package.

## Results

### Baseline characteristics

A total of 80 AMI patients and 36 healthy controls were included to explore the feasibility of HULC and TTTY15 as novel biomarkers to diagnose AMI, with an average age of 58.50 and 58.25, respectively. No significant difference could be observed in age, the occurrence of diabetes mellitus, alcohol drinking, and cholesterol level between the two groups (*P* > 0.05). There was a significant difference in BMI (24.15 ± 2.29 versus 26.08 ± 2.06), the occurrence of hypertension (38.9% versus 67.5%), smoking population (46.1% versus 70.0%), total cholesterol level (4.22 ± 0.75 versus 4.54 ± 0.77), LDL level (2.76 ± 0.37 versus 3.34 ± 0.33), CK-MB level (53.97 ± 47.17 versus 108.39 ± 64.48) and troponin T level (0.04 ± 0.02 vs. 1.15 ± 0.56) between two groups (*P* < 0.05). Detail was documented in Table 1. For the verification cohort, the average age was 57.49 in the control group and 58.02 in the AMI group. Other baseline characteristics for the overall and verification cohort were shown in Additional file 2: Table S1.

**Table 1:** Baseline characteristics of AMI patient group and control group

	Control group (n = 36)	AMI group (n = 80)	*P* value
Age (years)	58.50 ± 4.29	58.25 ± 8.99	0.874
BMI (kg/m^2^)	24.15 ± 2.29	26.08 ± 2.06	< 0.001
Hypertension (n)	14 (38.9%)	55 (67.5%)	0.002
Diabetes mellitus (n)	16 (44.4%)	50 (58.8%)	0.069
Alcohol drinking (n)	16 (44.4%)	53 (66.3%)	0.027
Smoking (n)	13 (46.1%)	56 (70.0%)	< 0.001
Tc (mmol/L)	4.22 ± 0.75	4.54 ± 0.77	0.039
LDL (mmol/L)	2.76 ± 0.37	3.34 ± 0.33	< 0.001
HDL (mmol/L)	1.24 ± 0.12	1.15 ± 0.16	0.003
CKMB (U/L)	53.97 ± 47.17	108.39 ± 64.48	< 0.001
TnT (μg/L)	0.04 ± 0.02	1.15 ± 0.56	< 0.001

Values are presented in mean ± standard deviation (sd) or n(%)

*BMI* body mass index, *Tc* total cholesterol, *LDL* low density lipoprotein, *HDL* high density lipoprotein; *CK-MB* creatine kinase-MB, *TnT* troponin T

### Plasma level of lncRNA TTTY15 and HULC

To verify the possibility of lncRNA TTTY15 and HULC being novel biomarkers in AMI diagnosis, their plasma levels in AMI patients were tested in comparison with healthy control. The difference between the group was tested employing Mann Whitney test, including lncRNA level at admission (pre-PCI) versus post-PCI and lncRNA level at pre-PCI versus post-PCI.

**Fig. 1:**
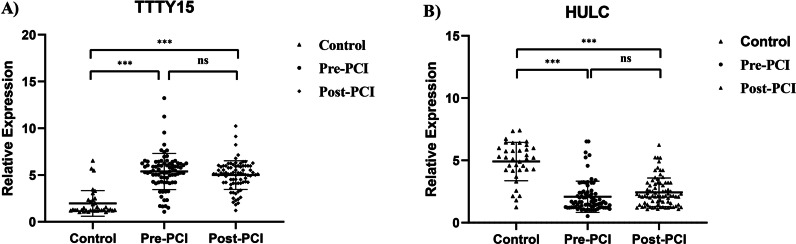
LncRNA relative expression level of TTTY15 and HULC between AMI patients and healthy controls in the discovery cohort. **A** Relative expression level of TTTY15 with the comparison of control, pre-PCI and post-PCI procedure; **B** Relative expression level of HULC with the comparison of control, pre-PCI and post-PCI. ***indicated *P* < 0.001 and ns indicated no significance

Regarding plasma TTTY15 level, a total of 80 AMI patients’ plasma and 36 healthy controls’ plasma were collected. After detecting the TTTY15 level via qRT-PCR calculated by 2^−ΔΔcq^ methods, TTTY15 level of AMI patients were significantly up-regulated in comparison with the control group, and detailed information was displayed in the scatter plot of Fig. 1A. For plasma HULC level, the same number of blood samples was tested. Inversely, HULC levels in AMI patients were significantly down-regulated in comparison with the control group, with scatter plot shown in Fig. 1B. The expression levels of two lncRNA in the verification cohort were shown in Additional file 3: Figure S2.

### The predictive power of lncRNA TTTY15 and HULC as novel biomarkers

**Fig. 2:**
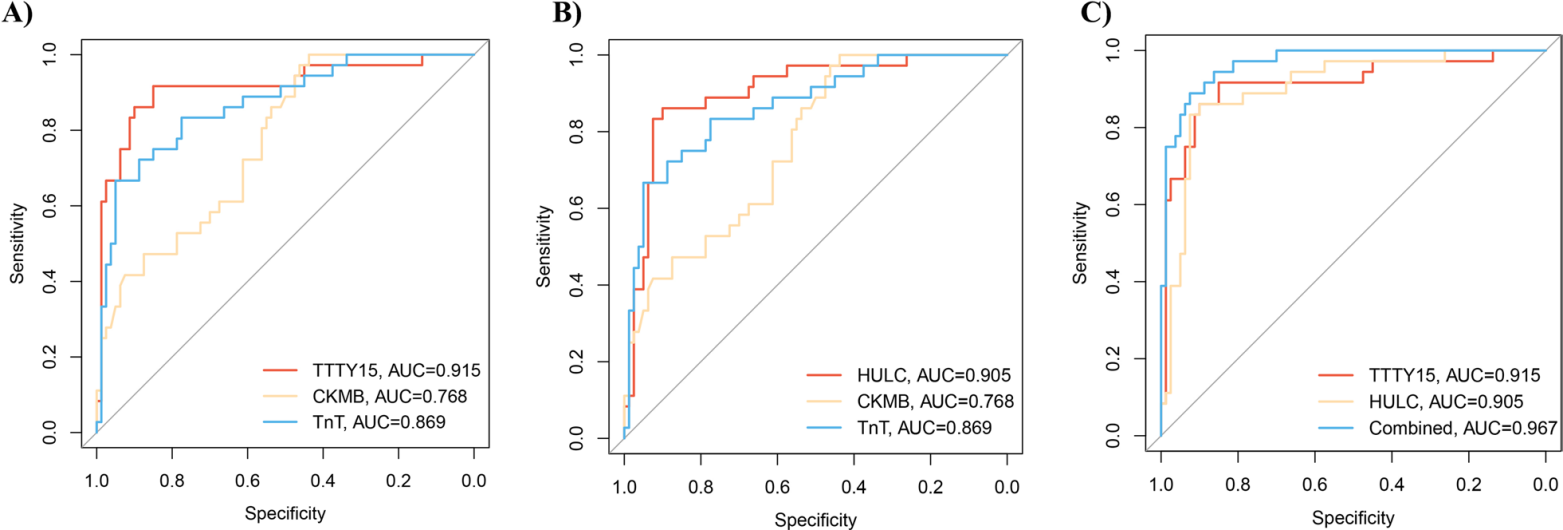
AUC of selected lncRNA. **A** ROC curve of TTTY15 (AUC = 0.915) in comparison with CKMB (AUC = 0.768) and TnT (AUC = 0.869); **B** ROC curve of HULC (AUC = 0.905) in comparison with CKMB (AUC = 0.768) and TnT (AUC = 0.869); **C** ROC curve showing the TTTY15 (AUC = 0.915), HULC (AUC = 0.905) and combination of TTTY15 with HULC (AUC = 0.967). *AUC* Area under the curve; *ROC curve* receiver operator characteristic curve

To evaluate the predictive power of TTTY15 and HULC as the novel biomarkers in AMI diagnosis, the ROC curve was preferentially adopted in verification. From Fig. 2A, ROC analysis unveiled the superiority of TTTY15 as biomarkers against conventional AMI biomarkers CKMB and TnT (AUC of TTTY 15: 0.915 versus CKMB: 0.768 versus TnT: 0.869). Similarly, as the results obtained from Fig. 2B, ROC analysis revealed the significant predictive power of HULC compared with CKMB and TnT (AUC of HULC: 0.905 versus CKMB: 0.768 versus TnT: 0.869). We also established a model with the combination of TTTY15 with HULC to test whether the predictive power would improve. As shown in Fig. 2C, the combination model revealed higher predictive power than TTTY15 or HULC alone (AUC of combination = 0.967).

**Fig. 3:**
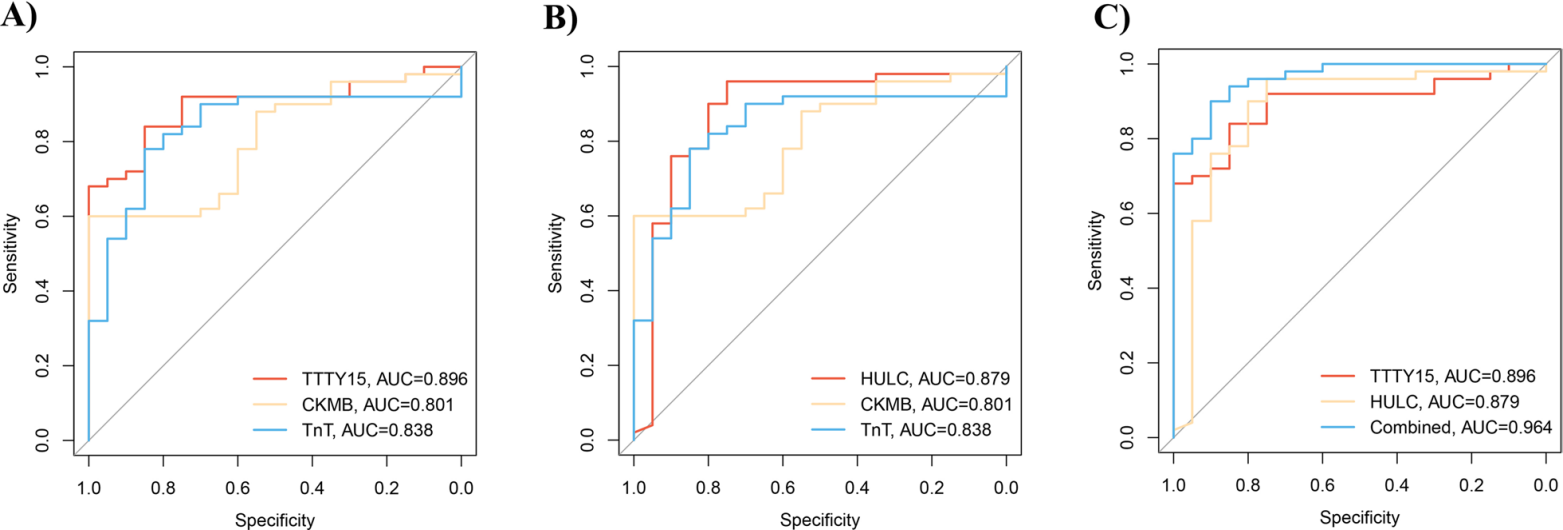
AUC of selected lncRNA in verification cohort. **A** ROC curve of TTTY15 (AUC = 0.896) in comparison with CKMB (AUC = 0.801) and TnT (AUC = 0.838); **B** ROC curve of HULC (AUC = 0.879) in comparison with CKMB (AUC = 0.801)and TnT (AUC = 0.838); **C** ROC curve showing the TTTY15 (AUC = 0.896), HULC (AUC = 0.879) and combination of TTTY15 with HULC (AUC = 0.964). *AUC* Area under the curve, *ROC curve* Receiver operator characteristic curve

Also, to test the potential diagnostic value of the selected lncRNA, AUC was tested in a verification cohort. As shown in Fig. 3, TTTY15 (AUC = 0.896, Fig. 3A) and HULC (AUC = 0.879, Fig. 3B) were revealed to be better biomarkers in comparison with CKMB (AUC = 0.801) and TnT (AUC = 0.838). Similarly, the combination of TTTY15 and HULC shows higher predictive power (AUC = 0.964, Fig. 3C). Further, to verify the predictive power of the selected lncRNA, we combined the exsiting biomarkers in the ROC analysis, indicating that predictive power of the selected lncRNA were higher than the exsiting biomarkers (Fig. 3 and Additional file 4: Figure S3).

Collectively, lncRNA TTTY15 and HULC are potentially valuable biomarkers in AMI diagnosis.

### Logistic regression and correlation analysis

To further validate the fidelity of our postulation, logistic regression was performed to investigate the contribution of selected lncRNA to AMI in cooperation with established risk factors. From logistic regression, widely accepted risk factors such as CKMB (OR 1.02, *P* < 0.001), TnT (OR 9.481, *P* < 0.001), and BMI (OR 1.487, *P* < 0.001) were significantly associated with AMI. In concert with established risk factors, lncRNA TTTY15 (OR 2.752, *P* < 0.001) and HULC (OR 0.358, *P* < 0.001) were revealed to be associated with AMI. Detailed information on logistic regression was documented in Table 2.

**Table 2:** Logistics regression analysis for the association of lncRNA TTTY15, HULC and potential risk factors with occurrence of AMI

Variable	B	S.E	Wald	*P*	OR	95% CI	
TTTY15	1.012	0.187	29.174	< 0.001	2.752	1.906	3.974
HULC	− 1.027	0.193	28.411	< 0.001	0.358	0.245	0.522
Age	− 0.043	0.029	2.191	0.139	0.958	0.904	1.014
DM	− 0.489	0.435	1.262	0.261	0.613	0.261	1.439
Tc	0.576	0.304	3.594	0.058	1.779	0.981	3.225
CKMB	0.015	0.004	14.166	< 0.001	1.015	1.007	1.023
TnT	2.249	0.468	23.082	< 0.001	9.481	3.787	23.733
BMI	0.397	0.111	12.754	< 0.001	1.487	1.196	1.849
Smoking	− 0.358	0.428	0.873	0.357	0.524	0.258	1.643
Alcohol	0.254	0.63	0.289	0.652	1.285	0.531	3.275

*DM* diabetes mellitus, *Tc* total cholesterol, *LDL* low density lipoprotein, *CKMB* creatine kinase isoenzymes, *TnT* troponin T, *BMI* body mass index

In addition, correlation analysis was performed to investigate the relationship between selected lncRNA and cardiovascular risk factors in the AMI group. For TTTY15, it was unveiled to be positively related to LDL (coefficient of 4.260, *P* < 0.008), CKMB (coefficient of 0.015, *P* < 0.001), and TnT (coefficient of 2.161, *P* < 0.001). To be in concordance with qRT-PCR results, HULC was revealed negatively related to CKMB (r = − 0.823, *P* < 0.001) and TnT (coefficient of − 1.115, *P* < 0.005). Detailed information about correlation analysis was shown in Table 3.

**Table 3:** Correlation analysis investigating the association between lncRNA TTTY15 and HULC with potential cardiac risk factors

	TTTY15		HULC	
	Coefficient	*P* value	Coefficient	*P* value
Age	− 0.039	0.259	0.040	0.135
BMI	0.352	0.002	− 0.247	0.005
Tc	0.576	0.058	− 0.465	0.085
LDL	4.260	< 0.001	− 12.611	< 0.001
CKMB	0.015	< 0.001	− 0.823	< 0.001
TnT	2.161	< 0.001	− 1.115	< 0.001

*BMI* body mass index, *Tc* total cholesterol, *LDL* low density lipoprotein, *CKMB* creatine kinase isoenzymes, *TnT* troponin T

## Discussion

Incremental studies have reported the potency of lncRNA being novel biomarkers to diagnose AMI, which may increase not only the accuracy but also the efficiency of the diagnostic process [17–19]. Meanwhile, lncRNA predominates in pivotal biological processes involved in cardiovascular diseases through ceRNA regulated gene expression, so exploration of these biomarkers is also beneficial to unveil potential therapy targets and get familiar with the underlying pathological changes of the myocardium [20, 21]. More importantly, lncRNA owns acceptable stability in peripheral circulation and detection sensitivity [22, 23]. Collectively, employing lncRNA plus conventional biomarkers is pressing needed in AMI diagnosis.

In the current study, we analyzed the lncRNA TTTY15 and HULC levels in peripheral blood of AMI patients and healthy volunteers to identify their potential capacity to be adopted as novel biomarkers in AMI diagnosis. Our results recapitulated that TTTY15 and HULC were plausible biomarkers to diagnose AMI. Interestingly, TTTY15 was upregulated while HULC was downregulated in AMI patients. ROC analysis delineated acceptable predictive power of TTTY15 and HULC as biomarkers compared with CKMB and TnT in AMI diagnosis. Moreover, consistent with established cardiovascular risk factors, TTTY15 and HULC levels were identified to be associated with AMI and, they have also correlated to LDL as well as CKMB levels. This is the first study revealing the capacity of lncRNA TTTY15 and HULC to be administered in AMI diagnosis, which may inspire cardiologists in clinical practice. A single biomarker has its inherent limitation in diagnosis, yet multi-biomarkers are available to provide more information about specific pathological mechanisms [24, 25]. Consequently, the combination of lncRNA as biomarkers in clinical practice is necessary and it could hint at an effective therapeutic strategy.

In previous studies, TTTY15 has been found highly expressed in peripheral blood of AMI patients and H_2_O_2_-stimulated AC16 cells model, which are in concert with our results [15, 26]. Moreover, TTTY15 downregulation or silence suppresses H_2_O_2_-stimulated AC16 cell apoptosis, inflammatory response, and oxidative stress, and improves cell viability. Based on these theories, not surprisingly, silenced TTTY15 could reduce the size of infarction in the AMI model [15]. Apart from being a potential biomarker in AMI diagnosis, the abovementioned experimental evidence extrapolates that suppressing TTTY15 could be a plausible pathway to alleviate the progression of AMI, providing novel therapeutic targets of treatment.

Before being acknowledged the roles in the cardiovascular field, lncRNA HULC has been studied thoroughly in the cancer domain. It has been reported to be the novel biomarkers in hepatocellular carcinoma and abnormally expressed in pancreatic and gastric cancer [27–29]. Recently, HULC has been explored its function in the cardiovascular field. It has been revealed that HULC would be downregulated in the I/R-injured myocardial model and overexpressed by miR-377-5p mediation leading increase of conventional myocardial injury biomarkers (Troponin-T and CKMB) level in the H9c2 cell model [14]. In addition, HULC plays an essential role in cellular inflammation, a pivotal pathway involved in myocardial injury. Knockdown of HULC has been found to profoundly reduce inflammatory factors level (IL-6, ICAM1, VCAM1) in lipopolysaccharide (LPS) treatment induced cellular experiments, and its overexpression could significantly relieve TNF-α induced cell injury [30, 31]. Collectively, in combination with our results, HULC could be not only the diagnostic biomarkers but also the potential therapeutic target.

Several limitations should be considered in this research. Firstly, although the positive results delineate the potentially novel biomarkers in AMI diagnosis, future studies investigating these lncRNAs with larger sample sizes are imminent. Secondly, lncRNA TTTY15 and HULC are associated with other diseases so the administration of these novel biomarkers should rule out the confounding diseases. Thirdly, qRT-PCR is the preferential method to detect RNA while it is expensive and time-consuming, so quantification of lncRNA in clinical practice is challenged. Last but not the least, it should be noted that the pathophysiology of the lncRNA should be further studied due to the currently limited knowledge.

## Conclusion

Our results recapitulate that lncRNA TTTY15 and HULC are significantly different between AMI patients and healthy controls, revealing the potential capacity of these lncRNAs to be novel biomarkers to assist AMI diagnosis with the combination of conventional biomarkers, while the relatedly confounding diseases should be ruled out when administering these biomarkers.

## Supplementary Information

**Additional file 1.** Cq value or each group.

**Additional file 2.** Baseline characteristics of the verification cohort.

**Additional file 3.** LncRNA relative expression level in the verification cohort.

**Additional file 4.** AUC of the selected lncRNA and existed biomarkers.

## Data Availability

The dataset supporting the conclusions of this article is included within the article. There was no any sequencing, genomic, phylogenetic data generated during this study. Request for the related data of this study should refer to Dr. Xie, the corresponding author.
