# Supplementary material for: Circulating long non-coding RNA TTTY15 and HULC serve as potential novel biomarkers for predicting acute myocardial infarction
Source: BMC Cardiovasc Disord. 2022 Mar 4;22:86. doi: 10.1186/s12872-022-02529-5 (PMC8895090; doi:10.1186/s12872-022-02529-5)
Supplement: Supplementary file 2 — Additional file 2. Baseline characteristics of the verification cohort. [file 12872_2022_2529_MOESM2_ESM.docx]

**Table S1. Baseline Characteristics of the verification cohort**

|  | Control group (n=20) | AMI group (n=50) | P value |
| --- | --- | --- | --- |
| Age (years) | 57.49±4.23 | 58.02±4.45 | 0.650 |
| BMI (kg/m^2^) | 23.47±2.46 | 25.45±2.38 | 0.003 |
| Hypertension (n) | 7 (35.0%) | 34 (68.0%) | 0.011 |
| Diabetes Mellitus (n) | 3 (15.0%) | 27 (54.0%) | 0.003 |
| Alcohol drinking (n) | 11 (55.0%) | 29 (58.0%) | 0.819 |
| Smoking (n) | 8 (40.0%) | 32 (64.0%) | 0.067 |
| Tc (mmol/L) | 4.01±0.54 | 4.36±0.73 | 0.057 |
| LDL (mmol/L) | 2.65±0.41 | 3.32±0.37 | <0.001 |
| HDL (mmol/L) | 1.19±0.17 | 1.21±0.14 | 0.614 |
| CKMB (U/L) | 52.19±40.28 | 118.34±55.78 | <0.001 |
| TnT (μg/L) | 0.12±0.05 | 1.14±0.62 | <0.001 |

Values are presented in mean±standard deviation (sd) or n(%).

Abbreviations: BMI, Body Mass Index; Tc, Total cholesterol; LDL, Low Density Lipoprotein; HDL, High Density Lipoprotein; CK-MB, Creatine Kinase-MB; TnT, Troponin T.
