# Supplementary figures and images for: Circulating long non-coding RNA TTTY15 and HULC serve as potential novel biomarkers for predicting acute myocardial infarction
Source: BMC Cardiovasc Disord. 2022 Mar 4;22:86. doi: 10.1186/s12872-022-02529-5 (PMC8895090; doi:10.1186/s12872-022-02529-5)

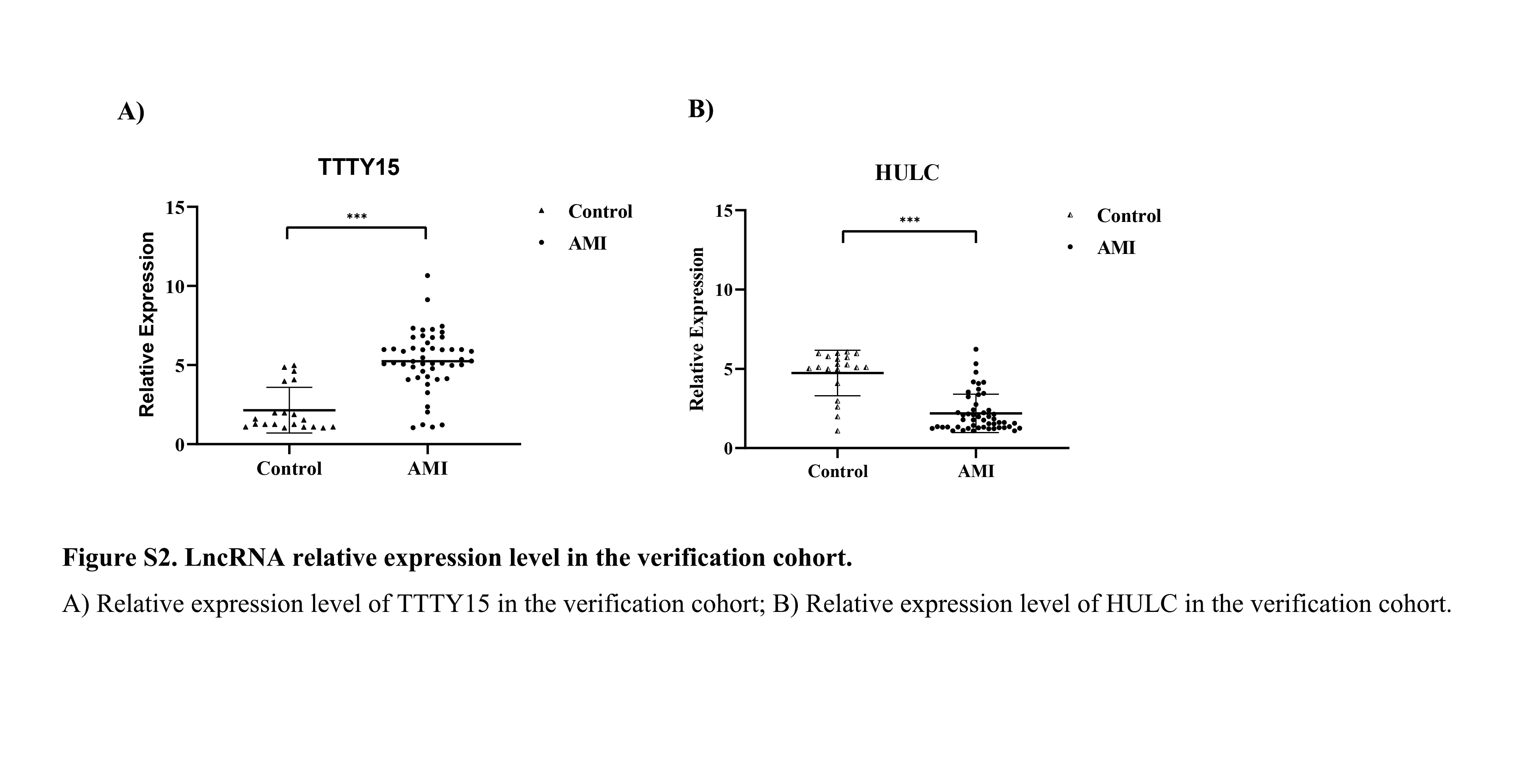

Supplement: Supplementary file 3 — Additional file 3. LncRNA relative expression level in the verification cohort. [file 12872_2022_2529_MOESM3_ESM.jpg]
